# Mixed-type autoimmune hemolytic anaemia complicated by acute cerebral infarction: a case report

**DOI:** 10.3389/fmed.2025.1587960

**Published:** 2025-08-12

**Authors:** Lei Wang, Hui Li, Hao Zhao, Zhonglin Wang

**Affiliations:** ^1^Shandong University of Traditional Chinese Medicine, Jinan, China; ^2^Emergency Department, Affiliated Hospital of Shandong University of Traditional Chinese Medicine, Jinan, China

**Keywords:** acute cerebral infarction, autoimmune hemolytic anemia, case report, mixed-type, warm and cold autoantibodies

## Abstract

**Background:**

In patients with autoimmune hemolytic anaemia (AIHA), numerous factors can influence disease severity, and thrombotic complications are associated with increased morbidity and mortality. Reports of autoimmune hemolytic anemia complicated by acute cerebral infarction are relatively rare.

**Case presentation:**

We report a case of an 82-year-old female patient with hypertension who developed mixed-type AIHA complicated by acute cerebral infarction following intravenous infusion of ceftriaxone after erysipelas in which the patient’s previous hemoglobin (Hb) level was maintained at approximately 108 g/L. After developing erysipelas and treatment with ceftriaxone, the patient experienced a continuous decline in Hb, hematocrit (HT), and red blood cell (RBC) counts. Direct antiglobulin test (DAT) was positive for IgG and C3d, and both the reticulocyte count and proportion were elevated. The treatment regimen included methylprednisolone, enoxaparin for anticoagulation, and clopidogrel for antiplatelet aggregation. Following targeted treatment, the patient’s condition improved.

**Conclusion:**

Clinicians should be aware of the potential contributing factors in patients with mixed-type AIHA who develop neurological deficits due to severe anemia. This case report aims to emphasize the laboratory aspects of mixed-type AIHA and the necessity for clinicians to recognize the potentially fatal consequences of acute thromboembolism in mixed-type AIHA.

## Introduction

Autoimmune hemolytic anemia (AIHA) is an acquired, heterogeneous group of diseases that includes warm AIHA, cold agglutinin disease (CAD), mixed-type AIHA, paroxysmal cold hemoglobinuria and atypical AIHA (1). The hallmark of AIHA is an immune dysregulation in the body’s B lymphocytes, which mistakenly identifies the surface antigens of its own red blood cells as foreign and produces antibodies against them, leading to the destruction of red blood cells, resulting in hemolysis and anemia. Complement activation is a pathogenic hallmark of hemolytic anemia. Hemolysis occurs when antigen-negative red blood cells (RBCs) are lysed by the complement system. This mechanism of bystander erythrocyte destruction is incorporated into multiple clinical entities, including passenger lymphocyte syndrome, post-transfusion hyperhemolysis, and paroxysmal nocturnal hemoglobinuria (PNH) (2). In patients with AIHA, multiple factors may influence disease severity. Acute exacerbations of AIHA may signify an intricate pathophysiological link between intravascular hemolysis and consequent thrombosis, with thrombotic complications associated with elevated morbidity and mortality. Mixed-type AIHA, defined by the concurrent presence of warm and cold autoantibodies, demonstrates greater complexity in clinical manifestations and unpredictability of therapeutic responses. Reports of AIHA complicated by acute cerebral infarction are rare. This article presents a case of an 82-year-old female patient with hypertension who developed mixed-type AIHA complicated by acute cerebral infarction following intravenous infusion of ceftriaxone after suffering from erysipelas. This case highlights an underrecognized association between mixed-type AIHA and ischemic cerebrovascular events, exemplifying the intricate management and fluctuating clinical course in such patients.

## Case presentation

**Figure 1:**
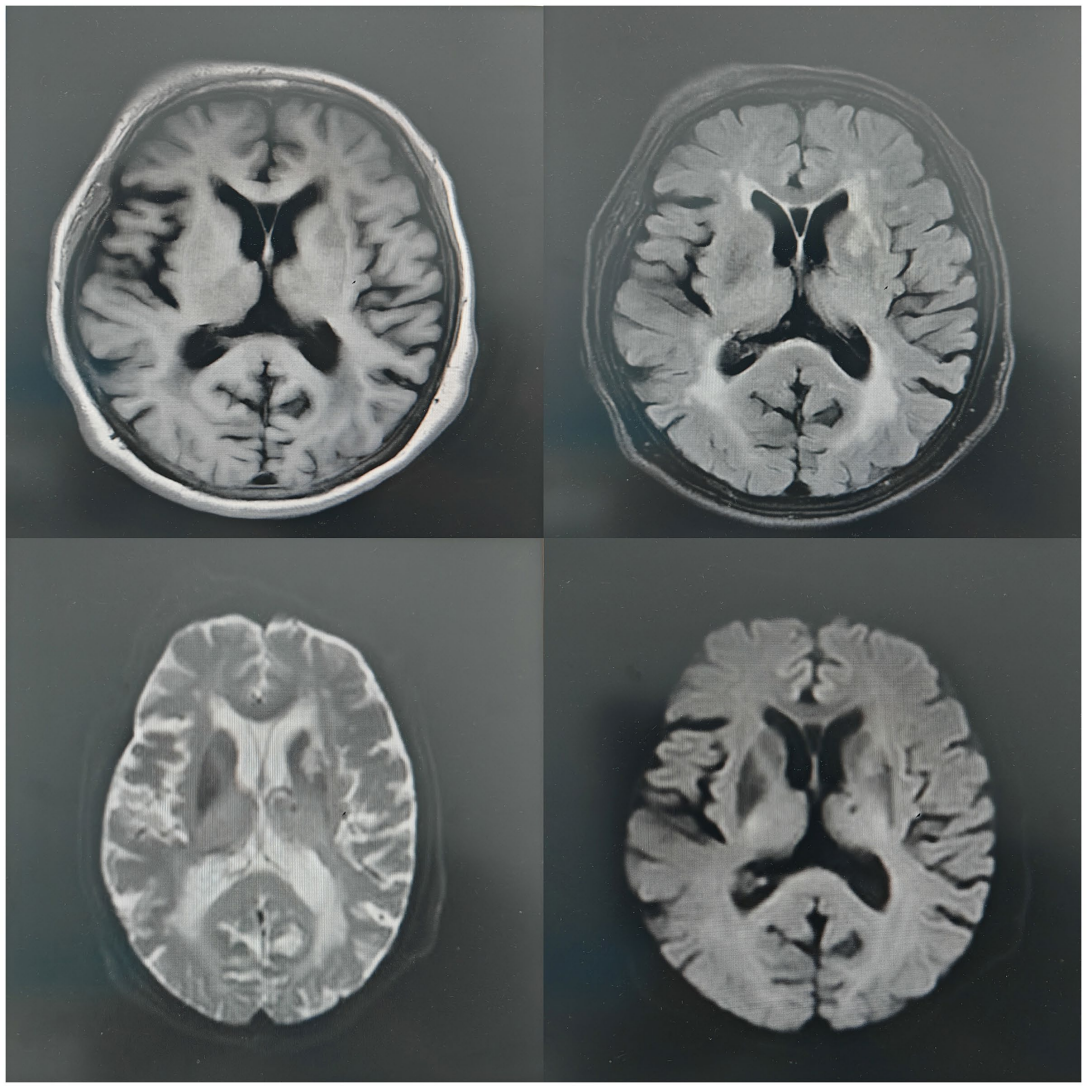
Brain magnetic resonance imaging showed a new cerebral infarction of the left radial crown.

An 82-year-old female with a history of hypertension was transferred to our hospital because of an unsteady gait for 3 days. The patient developed redness, swelling, and pain in her right lower limb 16 days prior to admission and sought treatment at a community hospital. Blood tests revealed a white blood cell count of 11.08 × 10^9^/L, absolute Neutrophil Count of 9.33 × 10^9^/L, neutrophil percentage of 84.2%, hemoglobin level of 108 g/L, and C-reactive protein level of 31.0 mg/L. She was diagnosed with erysipelas of the lower limb and treated with intravenous ceftriaxone. After 8 days of treatment, the symptoms of redness, swelling, and pain in the right lower limb improved, and the medication was discontinued. Three days prior to admission, the patient experienced unsteady gait but did not seek medical attention. One day prior to admission, she experienced a transient episode of loss of consciousness and was admitted to another hospital. Cranial magnetic resonance imaging (MRI) revealed new infarcts in the left semioval center and bilateral corona radiata (Figure 1). The patient was transferred to our hospital the following day for further treatment. During further history-taking, the patient reported an allergy to valsartan but denied any allergies to ceftriaxone or other cephalosporins. Physical examination showed a body temperature of 36.3°C, pulse rate of 78 beats/min, respiratory rate of 17 breaths/min, and blood pressure of 178/83 mmHg, pale palpebral conjunctiva, alert, appearance, Glasgow Coma Scale score of 15 (Eye opening: 4, Verbal: 5, Motor: 6), Modified Rankin Scale (mRS) score of 3, no dry or moist rales in either lung, and grade 4 muscle strength in the right limbs. Laboratory tests performed in the ward showed the following results: red blood cell count 1.97*10^12^/L, hemoglobin 61 g/L, reticulocyte count 147.70*10^9^/L, reticulocyte percentage 7.5%, blood glucose 8.5 mmol/L, lactate dehydrogenase 347 U/L, D-dimer 1.43 μg/mL, total bilirubin 28.6 μmol/L, indirect bilirubin 22.4 μmol/L, folate level 7.09 ng/mL, Serum iron level 13.9 μmol/L and vitamin B12 level 360.00 pg./mL. Doppler ultrasonography of the lower limbs revealed thrombosis of the right calf muscular vein. Computed tomography (CT) scan of the chest, abdomen, and pelvis revealed no evidence of enlarged lymph nodes. Echocardiography revealed no evidence of an intracardiac thrombus, while carotid Doppler and transcranial color-coded duplex (TCCD) examinations showed no stenosis in the cerebrovascular or carotid vascular systems.

**Figure 2:**
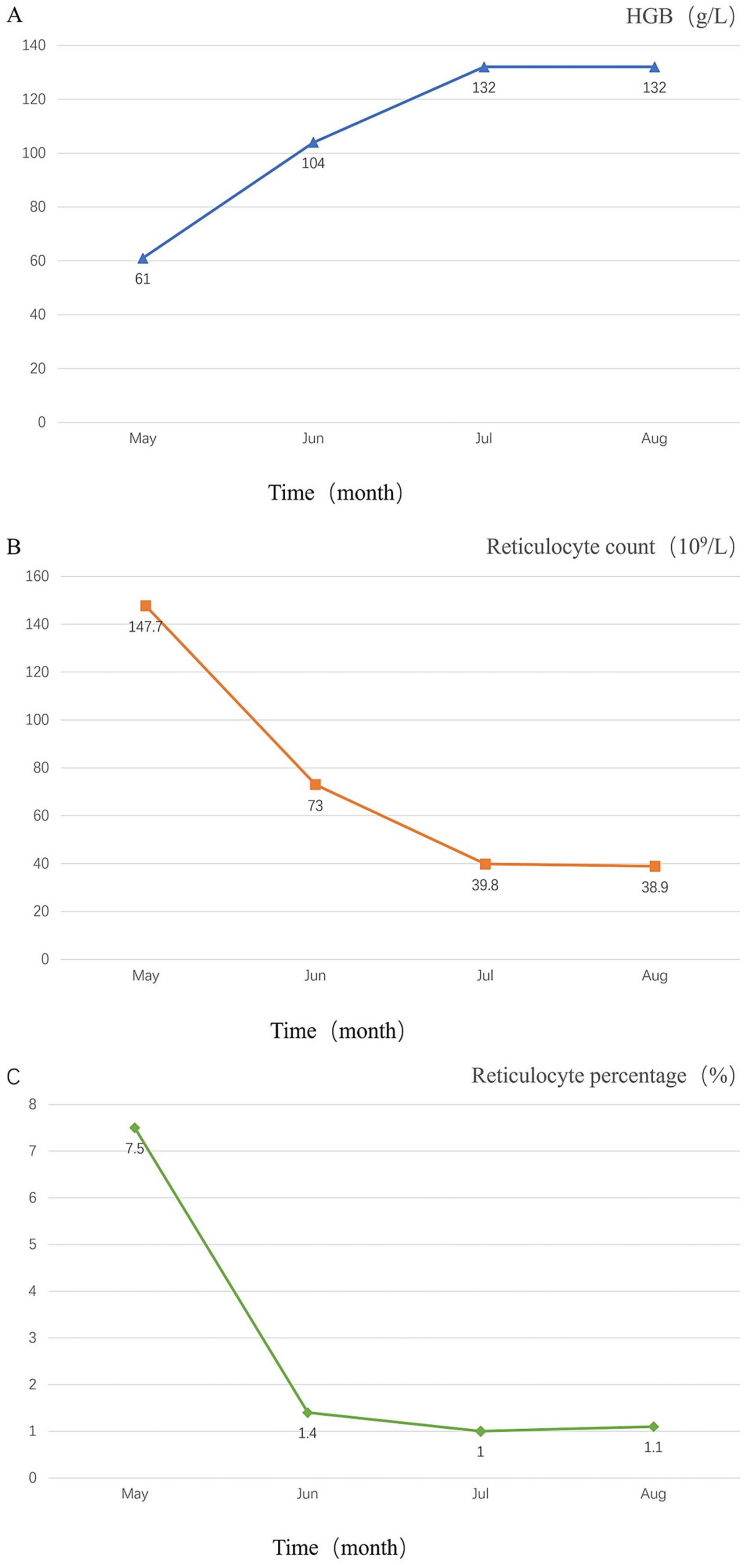
Temporal changes in the patient’s laboratory tests. **(A)** Temporal changes in hemoglobin levels; **(B)** temporal changes in reticulocyte count; **(C)** temporal changes in Reticulocyte percentage.

After consultation with the hematology department, the following laboratory tests were performed: DAT (+), blood and urine immunofixation electrophoresis (−), alpha-fetoprotein and carcinoembryonic antigen (−), ANA profile (−), and anti-neutrophil cytoplasmic antibody (−). Bone marrow biopsy results showed: mildly active erythroid hyperplasia, decreased myeloid-to-erythroid (M: E) ratio, predominance of late-stage erythroid precursors, and the presence of erythroid islands. Blood samples were sent to the Shandong Blood Center for immunohematological testing, which demonstrated a positive DAT for IgG and C3d. The cold agglutinin titer was 1:256 at 4°C, with a thermal amplitude extending to 32°C. The patient was diagnosed with mixed-type AIHA complicated by acute cerebral infarction and lower limb venous thrombosis. The treatment regimen includes methylprednisolone 40 mg once daily, enoxaparin for anticoagulation, and clopidogrel for antiplatelet aggregation. Subsequently, the patient’s condition improved, and hemoglobin levels gradually recovered. The patient was ultimately discharged after a 15-day hospitalization, with regained strength in the right limbs enabling cane-assisted ambulation (mRS score of 2). The discharge medications included methylprednisolone 32 mg QD (tapered gradually until discontinuation) and clopidogrel as antithrombotic therapy. LDH, bilirubin, and haptoglobin were tracked weekly until normalization. Two months later, a follow-up examination showed that the patient’s hemoglobin level was 132 g/L. Continued follow-up over six months revealed that the patient’s hemoglobin levels remained within the normal range. The patient was very satisfied with the treatment. The main laboratory test results and other auxiliary examinations during the diagnostic process are summarized in Table 1, and the temporal changes in laboratory tests of patients after hospitalization and follow-up after discharge are shown in Figure 2.

**Table 1:** Laboratory data at the onset of mixed-type AIHA.

Laboratory parameters	Results	Reference range
ANA profile	Negative	Negative
Albumin, g/L	33.5	40.0–55.0
ALT, U/L	27	7–40
ANCA	Negative	Negative
APTT, sec	35.4	28.0–43.5
AFP, ng/ml	1.62	≤7
ASO, IU/ml	459.0	0–408
AST, U/L	34	13–35
BUN, mmol/L	6.52	3.1–8.8
CRP, mg/L	11.70	0.00–8.00
CEA, ng/ml	1.32	≤5
Creatinine, μmol/L	44	41–81
D-dimer, μg/ml	1.43	0–0.50
Direct Antiglobulin Test	Positive	Negative
Folate, ng/mL	7.09	4.20–19.8
Fibrinogen, g/L	3.70	2.00–4.00
Hemoglobin, g/L	61	115–150
Hematocrit,%	16.4	35.0–45.0
INR, ratio	1.11	0.80–1.20
LDH, U/L	347	120–250
PT, sec	14.4	11.0–15.0
Red blood cell, 10^12^/L	1.97	3.80–5.10
Reticulocyte count, 10^9^/L	147.70	36.3–195.7
Reticulocyte percentage, %	7.5	0.5–1.5
Serum Iron, μmol/L	13.9	7.8–32.2
TP, g/L	74.3	65.0–85.0
Total bilirubin, μmol/L	28.6	0–21
Unconjugated bilirubin, μmol/L	22.4	0–17
Vitamin B12, pg./ml	360.00	197–771
White blood cell, 10^9^/L	9.48	3.5--9.5

TP = total protein; LDH = lactate dehydrogenase; ASO = Anti-Streptolysin O; INR = international normalized ratio; APTT = activated partial thromboplastin time; PT = Prothrombin Time; AFP = Alpha-fetoprotein; CEA = carcinoembryonic antigen; ANCA = anti-neutrophil cytoplasmic antibody.

## Discussion

In this case, the patient’s previous hemoglobin level was maintained at approximately 108 g/L. After administration of ceftriaxone for erysipelas, there was a continuous decline in Hb, hematocrit, and red blood cell counts. Bone marrow biopsy revealed mildly active erythroid hyperplasia with a decreased myeloid to erythroid ratio, predominantly consisting of cells at the late stages of maturation, and the presence of erythroid islands. DAT was positive for IgG and C3d. The stool occult blood test results were negative, and serum levels of vitamin B12, folate, and iron were within normal ranges, thereby excluding hemorrhagic anemia, iron deficiency anemia, megaloblastic anemia, and myelopathic anemia. These findings are consistent with the diagnostic criteria for AIHA. In this report, we describe the clinical manifestations and laboratory parameters of an 82-year-old female patient with mixed AIHA. AIHA represents a category of decompensated acquired hemolytic anemia caused by immune system dysregulation, with common secondary factors, including infections, medications, autoimmune diseases, and blood transfusions. Viral infections and other infectious diseases are recognized as causes of AIHA (3). Few case reports have identified hemolytic streptococcus as the causative agent of hemolytic anemia. The authors could not find any other case studies in the literature that reported hemolytic streptococcus as the etiology of mixed-type AIHA.

Infectious hemolytic anemia typically does not present with a significant decrease in hemoglobin levels and is self-limiting, with hemolytic symptoms improving as the infection is controlled. In this case, the patient’s infection was managed with ceftriaxone treatment; however, the Hb level continued to decline, which temporarily ruleds out infectious causes. The patient had no history of autoimmune disease, and the relevant tests during hospitalization were negative. Additionally, there was no history of blood transfusion prior to a drop in Hb level, which excluded autoimmune diseases and transfusion-related triggers. Ultimately, this patient had a previous exposure to ceftriaxone accompanied by a sharp decline in hemoglobin, necessitating a focus on excluding drug-induced factors. Drug-related immune hemolytic anemia is a rare condition with an incidence rate of 1 to 2 cases per 1,000,000 individuals annually. Its symptoms are insidious and easily overlooked in clinical practice, with a mortality rate as high as 40% (4). Currently, approximately 150 drugs are known to cause drug-related immune hemolytic anemia via various mechanisms. They are categorized into those induced by drug-independent antibodies (autoantibodies) and those induced by drug-dependent antibodies. The latter are activated only in the presence of the drug (or its metabolites) and represent the most common type of drug-related immune hemolytic anemia. Among these, cefotetan, ceftriaxone, and piperacillin have been frequently reported (5). Hemolytic anemia caused by ceftriaxone is characterized by intravascular hemolysis and features of an immune complex-mediated reaction. Following intravenous administration, ceftriaxone IgM antibodies can appear in red blood cells, forming drug-anti-drug antibody immune complexes that non-specifically bind to the red blood cell membrane, activate the complement system, and destroy red blood cells, ultimately leading to a hemolytic reaction. Immune hemolytic anemia induced by ceftriaxone is a rare but severe adverse reaction with non-specific clinical manifestations that are easily overlooked, potentially resulting in serious consequences. Once diagnosed with drug-related immune hemolytic anemia, patients should avoid re-exposure to the implicated drug, and the use of similar drugs should be approached with great caution.

AIHA leads to the destruction of red blood cells and a subsequent prothrombotic state, which may increase the risk of ischemic stroke (6). A retrospective study by Fattizzo et al. revealed that out of 287 patients with AIHA, 33 (11.4%) experienced thrombotic complications, including pulmonary embolism (*N* = 13), deep vein thrombosis of the lower extremities (*N* = 8), visceral vascular thrombosis (*N* = 2, comprising 1 case of portal vein thrombosis and 1 case of splenic vein thrombosis), superficial thrombophlebitis (*N* = 3), catheter-related thrombosis (*N* = 2), myocardial infarction (*N* = 3), and stroke (*N* = 2) (7). Another study indicated that the risk of thromboembolism is associated with an Hb level of 6 g/dL at the onset of the disease, intravascular hemolysis, and a history of splenectomy (8). In this patient, hemolytic anemia resulted in acute anemia and increased blood viscosity, further reducing cerebral blood flow and oxygen delivery, which contributed to neurological deficits.

A substantial body of literature has delineated the relationship between complement activation and thrombosis. Complement activation plays a substantial role in the prothrombotic tendency observed in patients with paroxysmal nocturnal hemoglobinuria (PNH). The complement and coagulation cascades are interconnected and function together during inflammation and hemostasis, which may ultimately lead to pathological thrombosis (9). Uncontrolled complement activation due to dysregulation of the alternative complement pathway observed in PNH, along with subsequent (intravascular) hemolysis, is considered to be the most thrombogenic condition (10). Hemolysis itself is a risk factor for thrombosis due to various mechanisms, including free heme released from damaged RBCs, exposure of phosphatidylserine on the RBC surface, circulating RBC-derived microparticles, endothelial impairment, stimulation of macrophages with increased procoagulant activity, and release of neutrophil extracellular traps (11). Moreover, complement inhibition might be beneficial for these patients based on recently published data. Gavriilaki et al. reported the first documented case of successful therapeutic application of the complement C3 inhibitor pegcetacoplan in warm autoimmune hemolytic anemia (wAIHA) (12).

AIHA treatment is divided into first-and second-line therapies. Glucocorticoids are the first-line treatment of choice, while second-line treatments include immunosuppressants and immunoglobulin pulse therapy (13). Most AIHA patients respond well to glucocorticoid treatment. However, if complicated by arteriovenous thrombosis, it can increase disability and mortality rates of patients. In this case, due to the patient’s advanced age, comorbidities, and resolution of hemolysis with conservative measures, immunosuppressive therapy was not initiated. AIHA combined with cerebral infarction is rare and can be easily overlooked. Therefore, as clinicians, it is crucial to pay attention to every detail of the patient’s condition changes, remain highly vigilant for possible neurological complications of the disease, and strive to avoid misdiagnosis and missed diagnosis to reduce the mortality rate of the disease.

## Conclusion

This patient had an acute cerebral infarction and was found to have severe anemia secondary to mixed-type AIHA. Mixed-type AIHA was likely triggered by the use of ceftriaxone following the diagnosis of erysipelas and clinicians should be aware of the potential contributing factors in patients with mixed-type AIHA who develop neurological deficits due to severe anemia. This case report aimed to emphasize the laboratory aspects of mixed-type AIHA and the necessity for clinicians to recognize the potentially fatal consequences of acute thromboembolism in mixed-type AIHA. Further studies should elucidate the pathophysiological mechanisms linking mixed-type AIHA with arterial ischemia, and develop appropriate treatment strategies for patients with concomitant cerebrovascular disease.

## Data Availability

The original contributions presented in the study are included in the article/supplementary material, further inquiries can be directed to the corresponding author.
